# Socioeconomic factors, sleep timing and duration, and amygdala resting-state functional connectivity in children

**DOI:** 10.3389/fpsyt.2024.1373546

**Published:** 2024-05-22

**Authors:** Melissa Hansen, Katrina R. Simon, Xiaofu He, Nick Steele, Michael L. Thomas, Kimberly G. Noble, Emily C. Merz

**Affiliations:** ^1^ Department of Psychology, Colorado State University, Fort Collins, CO, United States; ^2^ Department of Psychiatry, Columbia University Irving Medical Center, New York, NY, United States; ^3^ Molecular, Cellular and Integrative Neuroscience, Colorado State University, Fort Collins, CO, United States; ^4^ Department of Biobehavioral Sciences, Teachers College, Columbia University, New York, NY, United States

**Keywords:** socioeconomic disadvantage, sleep health, amygdala, functional magnetic resonance imaging, children

## Abstract

**Introduction:**

Reduced sleep health has been consistently linked with increased negative emotion in children. While sleep characteristics have been associated with neural function in adults and adolescents, much less is known about these associations in children while considering socioeconomic context. In this study, we examined the associations among socioeconomic factors, sleep duration and timing, and resting-state functional connectivity (rsFC) of the amygdala in children.

**Methods:**

Participants were typically-developing 5- to 9-year-olds from socioeconomically diverse families (61% female; *N* = 94). Parents reported on children’s weekday and weekend bedtimes and wake-up times, which were used to compute sleep duration and midpoint. Analyses focused on amygdala-anterior cingulate cortex (ACC) connectivity followed by amygdala-whole brain connectivity.

**Results:**

Lower family income-to-needs ratio and parental education were significantly associated with later weekday and weekend sleep timing and shorter weekday sleep duration. Shorter weekday sleep duration was associated with decreased amygdala-ACC and amygdala-insula connectivity. Later weekend sleep midpoint was associated with decreased amygdala-paracingulate cortex and amygdala-postcentral gyrus connectivity. Socioeconomic factors were indirectly associated with connectivity in these circuits via sleep duration and timing.

**Discussion:**

These results suggest that socioeconomic disadvantage may interfere with both sleep duration and timing, in turn possibly altering amygdala connectivity in emotion processing and regulation circuits in children. Effective strategies supporting family economic conditions may have benefits for sleep health and brain development in children.

## Introduction

Socioeconomic disadvantage during childhood is prevalent and linked with increased risk for mental health difficulties across the lifespan (1). Socioeconomic factors, such as family income and parental education, exert their effects on health and development through multiple mediating mechanisms (2), which are not fully understood. Altered sleep health during childhood likely plays a role in these mechanisms. Sleep health is a multifaceted construct encompassing multiple sleep parameters, such as sleep quantity, quality, and timing (3). Socioeconomic disadvantage has been repeatedly associated with shorter sleep durations and lower sleep quality in children (4–9). And, research using both experimental and correlational designs has linked disrupted sleep with an increased risk for mental health difficulties and altered emotion processing and regulation (10–13).

At the neural level, emotion processing and regulation, transdiagnostic factors underlying multiple psychiatric disorders (14), rely on neural networks involving the amygdala (15). In functional magnetic resonance imaging (fMRI) research, sleep duration and quality have been repeatedly linked with amygdala activation and functional connectivity in adults (16–19). Yet, far fewer studies have examined these associations in children. Previously, we proposed that socioeconomic disadvantage may lead to reduced sleep health in children, which may alter brain development in ways that increase risk for mental health problems (20). In the present study, we tested these ideas by examining the associations among socioeconomic factors, sleep duration and timing, and functional connectivity of the amygdala in children.

### Socioeconomic disparities in sleep duration and timing

In studies using actigraphy and parent-report sleep measures, socioeconomic disadvantage has been repeatedly associated with shorter sleep durations in children (4–9). Sleep timing refers to when sleep occurs and is often measured using bedtime, wake-up time, or the midpoint between sleep onset and wake-up. Later sleep timing has been related to delayed circadian functioning and sleep problems (21). Socioeconomic disadvantage has been frequently associated with later bedtimes in children (4, 22–26). Fewer studies have focused on sleep midpoint, which captures variability in both bedtime and wake-up time. In one study, socioeconomic disadvantage was associated with later sleep timing composite scores, which included sleep midpoint, during early childhood (27). In addition, preliminary correlations in two studies suggest that socioeconomic disadvantage may also be associated with later sleep midpoint in older children (28, 29).

Researchers have recommended considering sleep parameters jointly and on weekdays and weekends separately (30, 31). On weekdays, due to constraints on when children need to wake-up for school, later bedtimes often lead to shorter sleep durations. On weekends, children who go to bed later may be able to sleep in later, and in this way, get enough sleep; yet, their sleep timing is still later. While some previous studies have distinguished between weekday and weekend sleep timing (4, 22, 28), others have not (27, 29). Overall, few studies have considered sleep duration and timing simultaneously while distinguishing between weekdays and weekends. Thus, additional research is needed to gain a complete picture of how socioeconomic context may influence sleep health during childhood.

### Sleep duration and timing and emotional functioning

In clinical studies, insufficient sleep has been consistently associated with increased risk for anxiety and depressive disorders (10, 12, 13). These effects are likely due in part to the effects of disrupted sleep on multiple more specific, interrelated emotional processes, such as emotional reactivity and regulation (12, 32, 33). Experimental sleep restriction studies show that shorter sleep duration increases negative emotions and threat perception in adolescents and adults (32, 34–39). In a meta-review, shorter sleep duration was consistently associated with worse emotion regulation in children and adolescents (12).

Later sleep timing has also been linked with anxiety and depression in adults and adolescents (13, 40, 41) and with emotion regulation difficulties in children and adolescents (42). Yet, the associations of sleep duration and timing with neural function in emotion processing and regulation circuitry in children are not well understood.

### Sleep duration and timing and amygdala function

The amygdala is a subcortical structure in the limbic system that plays a crucial role in emotion processing and regulation, including threat detection and fear learning (15, 43, 44). Shorter sleep duration has been consistently associated with altered amygdala function in adults (11, 17, 32). More specifically, task-based fMRI studies have linked shorter sleep duration with increased amygdala reactivity to negative emotional stimuli in adults (19, 45) and children (46), and with greater amygdala activity during fear acquisition in adults (47). These results could stem in part from reduced prefrontal cortex (PFC)-mediated top-down control over the amygdala. Indeed, connections between medial PFC (mPFC) regions, such as the anterior cingulate cortex (ACC), and the amygdala have been found to support the downregulation of negative affect (15). Sleep deprivation has been associated with reduced amygdala functional connectivity with mPFC regions when viewing negative emotional stimuli (19, 32, 45, 48, 49). In addition, late chronotype, which is strongly associated with later sleep timing, has been associated with greater amygdala reactivity to fear faces compared to happy faces and reduced amygdala-dorsal ACC (dACC) task-based functional connectivity in adults (50).

### Sleep duration and timing and amygdala resting-state functional connectivity

Resting-state functional connectivity (rsFC) refers to the statistical association between time series of blood-oxygen-level dependent (BOLD) signal in distinct brain regions while a person is “at rest” or not performing a task (51, 52). In experimental studies, sleep deprivation and restriction have been associated with decreased rsFC between the amygdala and mPFC regions in adults (37, 53). In another study, shorter sleep duration the previous night was associated with increased amygdala rsFC with the ventromedial PFC in adults (18). Fewer studies of sleep duration and amygdala rsFC have focused on children. In one study, shorter self-reported weekend sleep duration was associated with lower rsFC between the amygdala and the superior temporal gyrus, ventral ACC, and precentral gyrus in children and adolescents (54).

Few studies have examined the associations between sleep timing and rsFC of the amygdala. In one study, later weekend sleep midpoint was associated with altered amygdala rsFC with the insula, dorsomedial PFC/dACC, supramarginal gyrus, postcentral gyrus, and superior frontal gyrus in children and adolescents (54).

Changes to amygdala rsFC in these circuits may partially explain the associations of shorter sleep duration and later sleep timing with emotional difficulties. Altered rsFC between the amygdala and mPFC has been linked to increased anxiety and depression symptoms and emotion regulation difficulties in children (55, 56). Understanding how sleep duration and timing may influence amygdala rsFC is essential to developing targeted and effective interventions that bolster mental health in children.

### Previous study

This study builds on our previous study of socioeconomic factors, sleep duration, and brain structure in the same sample of children. In that study, lower family income-to-needs ratio and parental education were significantly associated with shorter weekday sleep duration, which was significantly associated with reduced thickness in the middle temporal, postcentral, and superior frontal cortices and with smaller amygdala volumes (20). Here, we extend our previous work and make novel contributions to the literature by considering the role of multiple sleep factors and focusing on rsFC of the amygdala in children. This investigation builds upon the previous study by examining two indicators of sleep health – duration and timing – simultaneously, as recommended (30). In addition, our previous study used structural MRI (high-resolution T1-weighted MRI), whereas this study uses resting-state fMRI. Different MRI modalities offer complementary information about the brain’s organization (57).

### Current study

The goal of the current study was to investigate the associations among socioeconomic factors, sleep duration and timing, and rsFC of the amygdala in children. Participants were typically-developing 5‐ to 9‐year‐olds from socioeconomically diverse families. Parents reported on children’s weekday and weekend bedtimes and wake-up times, which were used to compute sleep duration and midpoint. Family income-to-needs ratio and parental education were examined separately, as they may reflect different aspects of children’s environments that have distinct effects on development (58) and sleep health (7, 59).

Based on prior findings (12, 16, 54) and the role of amygdala-ACC connectivity in emotion regulation (15), we hypothesized that shorter sleep durations and later sleep timing would be associated with altered connectivity between the amygdala and ACC in children. We also hypothesized that sleep duration and timing would mediate the associations between socioeconomic factors and amygdala-ACC connectivity in children. In addition, we examined whether sleep duration and timing were associated with connectivity between the amygdala and the rest of the brain, consistent with previous analytic approaches (37, 60, 61). Finally, we expected that sleep timing would be associated with amygdala rsFC independent of sleep duration.

## Methods

### Participants

Families were recruited through posting flyers, outreach, and local community events in New York, New York. Inclusionary criteria required families to be primarily English-speaking and children to be between 5 and 9 years of age and born from a singleton pregnancy with no history of premature birth, medical, or psychiatric issues. Families were excluded from the MRI portion of the study if children had contraindications for MRI scanning. Children ranged from 5.06 to 9.87 years of age (*N* = 94; 61% female). Fifty percent were reported to be Hispanic/Latinx; 31% African American, non-Hispanic/Latinx; and 14% European American, non-Hispanic/Latinx. Parental educational attainment ranged from 6.50 to 20.00 years, and family income-to-needs ratio ranged from.17 to 15.21, with family income ranging from $2,880 to $350,000.

Of the 48 children who participated in a resting-state fMRI scan, 41 had usable data, as described below. This fMRI subsample (*n* = 41) did not differ significantly in socioeconomic background from those without fMRI data.

### Procedure

Parents and their children made two visits to the lab within one month. During the first visit, parents provided written informed consent and then completed questionnaires asking about socioeconomic factors and their child’s weekday and weekend bedtime and wake-up time. Most families were invited to participate in the MRI portion of the study, which included a mock scan to acclimate children to the scanning environment. Full details of the MRI procedures for this study are provided in Merz et al. (62). During the second visit, children participated in an MRI scanning session which included a resting-state fMRI scan. The Institutional Review Boards at Teachers College, Columbia University and the New York State Psychiatric Institute approved this study.

### Measures

#### Socioeconomic factors

Parental educational attainment was computed as the average number of years of education across parents. Family income-to-needs ratio was calculated by dividing household income by the U.S. federal poverty line for the household size in the year of their participation in the study (63). To address positive skew, family income-to-needs ratio was log-transformed. Parental education and family income-to-needs ratio were significantly correlated (*r* = .68, *p* <.001; **Supplementary Table S1**).

#### Sleep duration and timing

Parents reported on their children’s bedtimes and wake-up times for a typical weekday and weekend day in the previous two weeks. Questions included “What is your child’s weekday bedtime?” and “When does your child wake up on weekdays?” Child sleep duration was computed separately for weekdays and weekends by calculating the time between bedtime and wake-up time (8, 64, 65). Sleep midpoint was calculated separately for weekdays and weekends as the point in time halfway between bedtime and wake-up time. Clock time was decimalized for these calculations (e.g., 8:30 PM = 8.5). Bedtime and wake-up time were not available for two children; therefore, the total sample size for sleep midpoint was 92. Following from our previous publication (20), only weekday (not weekend) sleep duration was analyzed in the current study; both weekday and weekend sleep midpoints were analyzed.

#### Parental anxiety and depression symptoms

Parental depressive symptoms were measured using the nine-item Patient Health Questionnaire (PHQ-9) (66), a self-report measure based on the diagnostic criteria for major depressive disorder. Parents indicated how often in the past two weeks they had depressive symptoms using a 4-point scale ranging from 0 (not at all) to 3 (nearly every day). Responses were summed to create a total score, with higher scores indicating greater depressive symptoms (α = .84). The PHQ-9 has well-established internal consistency, test–retest reliability, and validity (66, 67).

Parental anxiety symptoms were measured using the Beck Anxiety Inventory (BAI) (68), a 21-item self-report measure of physiological and cognitive anxiety symptoms. Parents indicated how much in the past week they were bothered by anxiety symptoms using a 4-point scale ranging from 0 (not at all) to 3 (severely). Responses were summed to create a total score, with higher scores indicating greater anxiety symptoms (α = .91). The BAI has strong internal consistency, test–retest reliability, and concurrent validity (68). A parental anxiety/depression composite score was created to use as a covariate in analyses by standardizing and averaging the PHQ-9 and BAI scores.

### Image acquisition

Imaging data were collected using a General Electric (GE) MR750 3T scanner with a 32-channel head coil. A 5-minute echo planar imaging (EPI) sequence was collected with the following parameters: repetition time (TR) = 2200 ms, echo time (TE) = 30 ms, flip angle = 90°, voxel size = 3.5×3.75×3.75 mm, matrix size = 64 × 64, 140 volumes, 6 dummy scans, FOV = 24 x 24 cm, 34 axial slices. The children received instructions to stay awake and keep their eyes open throughout the scanning session, and a fixation cross was displayed on the screen. When scheduling permitted, a second resting-state scan was obtained, resulting in a second scan for 38 of the 48 children who participated in a resting-state scan. A high‐resolution, T1‐weighted fast spoiled gradient echo scan was also acquired (sagittal acquisition; TR = 7.1 ms; TE = min full; inversion time [TI] = 500 ms; flip angle = 11°; 176 slices; 1.0 mm slice thickness; FOV 25 cm; inplane resolution = 1.0 by 1.0 mm).

### Image processing

Standard image preprocessing and first-level analyses were conducted using the CONN Toolbox (69). Functional data were realigned, unwarped, slice-time corrected, and scrubbed. Scans that were outliers based on head motion were detected using Artifact Detection Tools (ART) (integrated in the CONN Toolbox) based on a framewise displacement (FD) threshold above.9 mm or global BOLD signal changes above 5 standard deviations from the mean. Participants were subsequently excluded if they had more than 25% outlier scans. This process resulted in 7 participants with unusable data due to excessive motion and a final sample of 41 participants with usable resting-state fMRI data.

Functional MRI data were first co-registered to the T1 image using the Statistical Parametric Mapping version 12 (SPM12) co-registration procedure, and the T1 image was normalized into Montreal Neurological Institute (MNI) space and segmented into gray matter, white matter, and cerebrospinal fluid tissue classes using the SPM12 unified segmentation and normalization procedure (70). A Gaussian kernel of 8 mm full width at half maximum (FWHM) was used for smoothing. Six head motion parameters were used as regressors of no interest in the first-level analyses, and denoising was performed using the anatomical CompCor (aCompCor) method (71) to account for potential confounding physiological or motion effects in the BOLD signal. Data were then band-pass filtered (0.01–0.10 Hz) to minimize the influence of head-motion and low-frequency drift. Mean FD, an average of the 6 different motion parameters (three planes, and three rotations), was averaged over the time course of the scan and used as a covariate in second-level analyses. Mean FD was not significantly associated with sleep duration or midpoint. ROI masks for the left and right amygdala and ACC were generated using the Automated Anatomical Labelling atlas (72).

### Statistical analyses

Multiple linear regression analyses were conducted in R (version 4.1.1) to examine the associations of family-income-to-needs ratio and parental education with weekday and weekend sleep midpoint. Covariates in these analyses were child age and sex. Effect sizes (*η_p_^2^*) are presented, with values of.01,.06, and.14 indicating small, medium, and large effects, respectively (73).

#### Seed-to-seed analyses (ROI-to-ROI analyses)

The BOLD time series of each ROI (amygdala, ACC) was computed as the average of the time series of all its component voxels. Resting-state FC between two ROIs was calculated as the Fisher z-transformed correlation coefficient of their time series, and these values were extracted. Multiple linear regression analyses were then conducted in R with left/right amygdala-left/right ACC connectivity (4 connections total) as the dependent variables and weekday sleep duration and weekday/weekend sleep midpoint as the independent variables. To account for multiple comparisons, false discovery rate (FDR) corrections were applied to analyses using the p.adjust function in R (74).

#### Seed-to-whole-brain analyses

For each participant, Pearson’s correlation coefficients were calculated between the left/right amygdala time course and the time course of all other voxels in the brain. These subject-level maps were then Fisher z-transformed and used in a whole-brain linear regression as the dependent variable. Weekday sleep duration and weekday/weekend sleep midpoint were the independent variables. A threshold of voxel-wise *p* < 0.005 (uncorrected) and cluster-level *p* < 0.05 using FDR and family-wise error (FWE) corrections were used. Covariates in all rsFC analyses (both seed-to-seed and seed-to-whole brain analyses) were age, sex, parental education, and mean FD (75). Given that shorter weekday sleep duration was associated with smaller amygdala volume (20), we also examined whether associations of sleep duration and midpoint with amygdala rsFC remained significant after additionally controlling for amygdala volume.

#### Mediation model

We examined whether sleep duration and midpoint mediated the associations between socioeconomic factors and amygdala rsFC (**Supplementary Figure S1**). Mediation analyses were performed using the “mediation” package in R (76). First, two regression models were specified: the mediator model in which the mediator (sleep duration or midpoint) was regressed on the independent variable (parental education or family income‐to‐needs ratio) and the outcome model in which the outcome (amygdala rsFC) was regressed on the independent variable and mediator. The outputs of these two regression models served as the main inputs to the “mediate” function that computes the direct, indirect, and total effects of the mediation model. The significance of the mediated or indirect effect was tested using nonparametric bootstrapping methods (with 10,000 random samples) and 95% confidence intervals. Mediation was only tested when there was evidence of significant *a* and *b* paths (**Supplementary Figure S1**). For the *b* path, mediation was only tested if there were significant associations of sleep duration or midpoint with amygdala rsFC. Age, sex, and mean FD were included as covariates in these analyses.

## Results

### Descriptive statistics

Descriptive statistics are presented in **Table 1**, and zero-order correlations are presented in **Supplementary Table S1**. In the full sample, the average weekday sleep midpoint was 1:43 AM (range: 12:30 – 3:45 AM) and weekend sleep midpoint was 3:04 AM (range: 12:30 – 6:00 AM). Very similar patterns were observed in the fMRI subsample; the average weekday sleep midpoint was 1:48 AM (range: 12:30 – 3:00 AM) and weekend sleep midpoint was 3:08 AM (range: 12:45 – 6:00 AM). On average, children were within the recommended range of sleep duration for their age for both weekdays and weekends (**Table 1**) (77).

**Table 1:** Descriptive statistics for sleep duration and timing (*N* = 92).

	*M*	*SD*	Range
Weekday bedtime (decimalized time)	8.65	.72	7.00–11.00
Weekday wake-up time (decimalized time)	6.76	.63	5.00–9.50
Weekday sleep midpoint (decimalized time)	1.71	.54	.50–3.75
Weekday sleep duration (hours)	10.11	.80	8.00–12.50
Weekend bedtime (decimalized time)	9.95	1.24	7.00–12.00
Weekend wake-up time (decimalized time)	8.19	1.50	6.00–12.00
Weekend sleep midpoint (decimalized time)	3.07	1.21	.50–6.00
Weekend sleep duration (hours)	10.24	1.29	7.50–13.50

*M*, mean; *SD*, standard deviation.

Approximately 30% of families in the sample had their sleep data collected during the summer. Time of year (summer vs. school year) was not associated with weekday or weekend sleep duration (*p* = .50-.52) or weekday or weekend sleep midpoint (*p* = .33-.79) while controlling for child age and sex. Of note, older age was significantly associated with reduced weekday (*β* = -.29, *p* = .004) and weekend sleep duration (*β* = -.30, *p* = .004), but age was not significantly associated with weekday (*β* = .03, *p* = .78) or weekend sleep midpoint (*β* = .13, *p* = .12) while controlling for child sex and parental education.

### Amygdala connectivity patterns

**Supplementary Figure S2** presents the results of whole-brain one-sample *t* tests examining left and right amygdala connectivity. Similar to patterns previously reported for children in this age range (78), the left and right amygdala showed widespread positive functional connectivity with subcortical regions, including the contralateral amygdala, bilateral hippocampus, thalamus, and with cortical regions including the insula, somatosensory regions, temporal regions, ventromedial and orbitofrontal cortex, and ACC. Negative connectivity was found with occipital regions, superior parietal regions, posterior cingulate, and clusters in the dorsolateral PFC. Patterns of connectivity for the left and right amygdala were very similar.

### Socioeconomic factors and sleep midpoint

**Figure 1:**
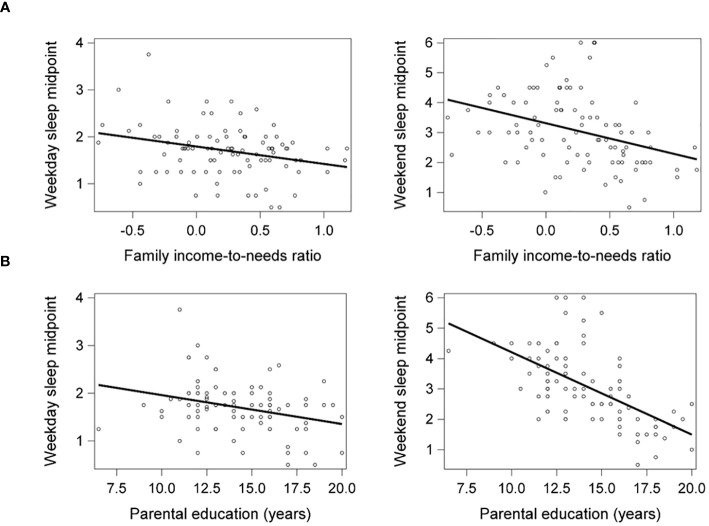
Lower family income-to-needs ratio (log-transformed) **(A)** and lower parental education **(B)** were significantly associated with later weekday and weekend sleep midpoints. Weekday and weekend sleep midpoint are shown in decimalized time.

Lower family income-to-needs ratio and parental education were significantly associated with later weekday (*β* = -.28, *p* = .009, *η_p_^2^* = .08; *β* = -.28, *p* = .009, *η_p_^2^* = .09, respectively) and weekend sleep midpoints (*β* = -.37, *p* <.001, *η_p_^2^* = .12; *β* = -.61, *p* <.001, *η_p_^2^* = .35, respectively) (**Figure 1**). Socioeconomic disadvantage was also significantly associated with shorter weekday sleep duration but not associated with weekend sleep duration, as reported previously (20).

### Sleep duration and midpoint and amygdala-ACC connectivity

**Figure 2:**
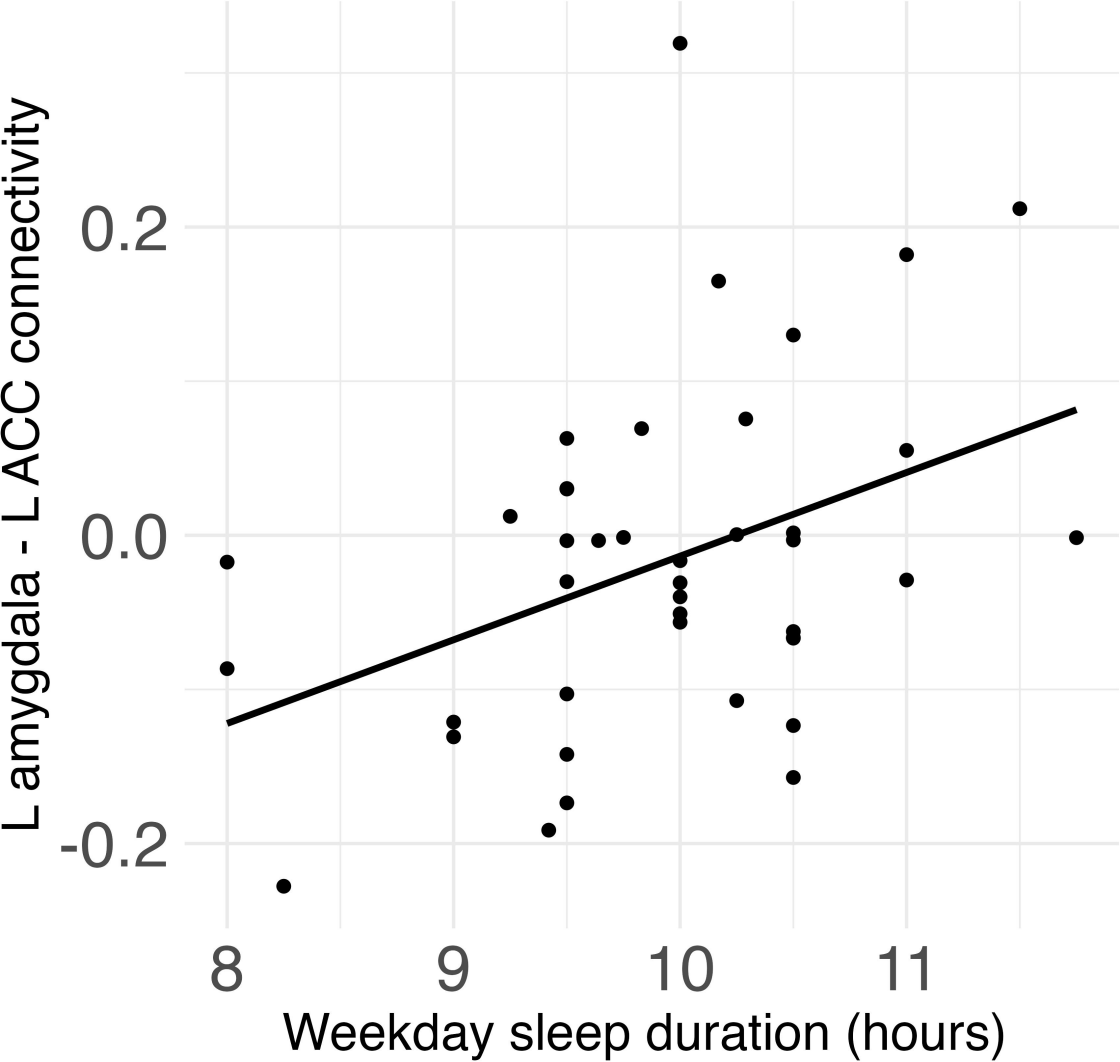
Weekday sleep duration was significantly positively associated with connectivity between the left amygdala and left anterior cingulate cortex (ACC) in children.

Weekday sleep duration was significantly positively associated with connectivity between the left amygdala and left ACC (*β* = .08, *FDR-corrected p* = .017) (**Figure 2**), and this association remained significant after additionally controlling for weekday sleep midpoint and amygdala volume. Neither weekday nor weekend sleep midpoint was associated with amygdala-ACC connectivity.

### Sleep duration and midpoint and amygdala whole-brain connectivity

**Figure 3:**
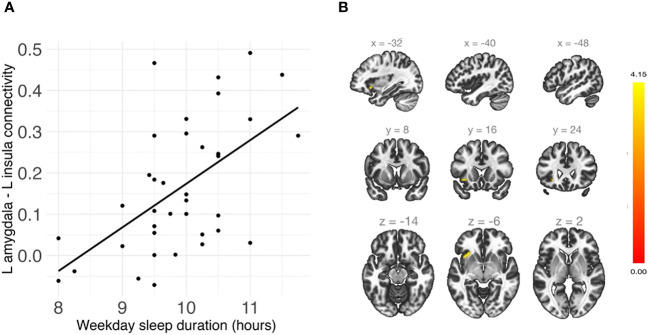
**(A)** Shorter weekday sleep duration was significantly associated with reduced connectivity between the left amygdala and left insula in children. **(B)** Sagittal, coronal, and axial views of the significant cluster in the insula.

**Figure 4:**
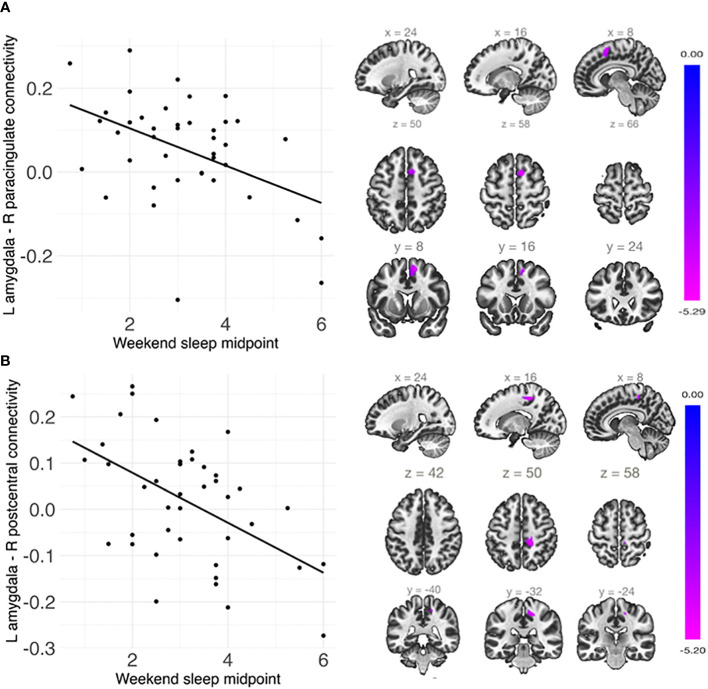
Weekend sleep midpoint was significantly negatively associated with connectivity **(A)** between the left amygdala and right paracingulate cortex and **(B)** between the left amygdala and right postcentral gyrus. Scatterplots are shown on the left, and sagittal, axial, and coronal views of the significant clusters are shown on the right. Weekend sleep midpoint is shown in decimalized time.

Shorter weekday sleep duration was significantly associated with decreased connectivity between the left amygdala and left insula (*β* = .07, *FDR-corrected p* = .048), and this association remained significant after additionally controlling for weekday sleep midpoint and amygdala volume (**Table 2** and **Figure 3**). In addition, weekend sleep midpoint was significantly inversely associated with connectivity between the left amygdala and right paracingulate gyrus (*β* = -.06, *FDR-corrected p* = .042) and the left amygdala and right postcentral gyrus (*β* = -.07, *FDR-corrected p* = .042) (**Table 2** and **Figure 4**), and these associations remained significant after additionally controlling for weekend sleep duration and amygdala volume. Weekday sleep midpoint was not significantly associated with amygdala whole-brain connectivity.

**Table 2:** Clusters that differed in their connectivity with the left amygdala as a function of sleep duration and midpoint.

Cluster	MNI coordinates			Size	p-FWE	p-FDR	Regions
	x	y	z				
Weekday sleep duration							
1	-28	+20	-8	80	.007	.048	Left insula, left frontal orbital cortex
Weekend sleep midpoint							
1	+12	+10	+56	197	.007	.042	Right paracingulate gyrus, right supplementary motor cortex, right postcentral gyrus
2	+16	-30	+50	167	.012	.042	Right postcentral gyrus, right precentral gyrus

FWE, family-wise error; MNI, Montreal Neurological Institute; FDR, false discovery rate. MNI coordinates are provided for the peak voxel, and cluster size indicates the number of voxels in the cluster.

### Socioeconomic factors, sleep duration and midpoint, and functional connectivity

Parental education was indirectly associated with connectivity between the left amygdala and left ACC (indirect or *ab* effect = .009, *p* = .02) and between the left amygdala and left insula (indirect or *ab* effect = .01, *p* = .02) via weekday sleep duration in children (**SupplementaryFigure S3**). These indirect effects were not significant for family income-to-needs ratio.

Parental education was indirectly associated with connectivity between the left amygdala and right paracingulate cortex (indirect or *ab* effect = .02, *p* <.001) and between the left amygdala and right postcentral gyrus (indirect or *ab* effect = .02, *p* <.001) via weekend sleep midpoint in children (**Supplementary Figure S4**). Similarly, family income-to-needs ratio was indirectly associated with connectivity between the left amygdala and right paracingulate cortex (indirect or *ab* effect = .07, *p* = .037) and between the left amygdala and right postcentral gyrus (indirect or *ab* = .07, *p* = .046) via weekend sleep midpoint.

### Sensitivity analyses

To further account for head motion (75, 79, 80), analyses were re-run using stricter thresholds for mean FD (81). Specifically, analyses were conducted after excluding children with mean FD > 1 mm (*n* = 5) and then excluding those with mean FD > 0.5 mm (*n* = 9). All results remained significant.

There was some variability in the time between sessions, with eight children who completed their MRI scan more than one month after their sleep data were collected. Thus, analyses were conducted to examine if the associations of sleep duration and midpoint with amygdala rsFC remained significant while additionally controlling for the time between sessions. All results remained significant (*p* = .02-.04). In addition, analyses were conducted to examine whether these associations remained significant after additionally controlling for parental anxiety and depression symptoms, similar to our previous publication (20). All results remained significant (*p* = .01-.04).

## Discussion

The goal of this study was to investigate the associations among socioeconomic factors, sleep duration and timing, and rsFC of the amygdala in 5- to 9-year-old children. Socioeconomic disadvantage was significantly associated with later sleep timing on both weekdays and weekends and, as reported previously (20), with shorter weekday sleep duration in children. Shorter sleep duration and later sleep timing were uniquely associated with altered amygdala connectivity in circuits associated with emotion processing and regulation. Socioeconomic disadvantage was indirectly associated with altered amygdala connectivity in these circuits via shorter weekday sleep duration and later sleep timing.

### Socioeconomic disadvantage is associated with later sleep timing in children

Lower family income-to-needs ratio and parental education were associated with later weekday and weekend sleep midpoints in children. These findings are consistent with those of previous studies linking socioeconomic disadvantage with later bedtimes in children (4, 22–26). Later sleep midpoint may suggest that sleep timing is misaligned with optimal functioning of the circadian system (28).

Together with our previous findings (20), these results suggest that socioeconomic disadvantage may interfere with both sleep timing and duration, with distinct patterns of effects on weekdays compared to weekends. Socioeconomic disadvantage may lead to later sleep timing across weekdays and weekends but shorter sleep duration only on weekdays. On weekdays, later bedtimes often lead to shorter sleep durations whereas on weekends, children may be able to compensate for later bedtimes by sleeping in later and consequently get enough sleep. The combination of frequently reduced sleep duration and later sleep timing during childhood could have negative effects on health and cognitive development. Both shorter sleep duration and later sleep timing have been associated with increased anxiety and depression (12, 40).

Socioeconomic disadvantage may lead to later sleep timing and shorter sleep duration in children because of lower-quality sleep environments (e.g., high noise levels, crowding, excess light, uncomfortable temperatures), greater stress, fewer routines, and more unpredictability (e.g., changes in parental work schedules) (6, 20, 82). Lower-quality sleep environments and inconsistent bedtime routines may lead to later bedtimes and difficulty falling asleep. Although our study focused on family socioeconomic factors, these associations may be due to both family- and neighborhood-level disadvantage. Previous work suggests that neighborhood factors may play an important role in sleep duration and timing (9, 25), and future research should disentangle the unique influences of family and neighborhood socioeconomic factors on children’s sleep characteristics.

### Sleep duration is associated with amygdala resting-state connectivity in children

Shorter weekday sleep duration was associated with decreased positive connectivity between the amygdala and ACC, independent of sleep timing. This result is consistent with previous studies of adults (37, 53) and children and adolescents (54). Developmentally, amygdala-mPFC rsFC may become increasingly positive across childhood (83). Increasingly positive amygdala-mPFC rsFC has been associated with stronger emotion regulation (55) and reduced aggressive behavior and attention problems in children (78). Thus, insufficient sleep duration may lead to patterns of amygdala-mPFC connectivity that make self-regulation more difficult for children.

Whole-brain analyses indicated that shorter weekday sleep duration was associated with reduced positive amygdala-insula connectivity, independent of sleep timing. The insula is critical for emotion generation and interoception (84). The amygdala has been found to show a normative pattern of positive rsFC with the insula in children (83). Although directionality varies, resting-state amygdala-insula connectivity has been associated with emotion regulation, anxiety and depression in children and adolescents (85–87). The anterior insula is a primary hub in the salience network (88), which is responsible for detecting and orienting to salient stimuli and coordinating neural resources, and the amygdala is also often included as part of this network (89, 90). Altered salience network function is thought to play a key role in the mechanisms underlying the effects of sleep deprivation on emotion processing (16, 32). Thus, these findings are consistent with previous work linking sleep duration with salience network connectivity. Decreased sleep duration may lead to functional uncoupling in circuits that facilitate emotion regulation and salience detection.

### Sleep timing is associated with amygdala resting-state connectivity in children

Later weekend sleep midpoint was associated with reduced connectivity between the amygdala and paracingulate cortex and between the amygdala and postcentral gyrus, independent of sleep duration. To our knowledge, only one resting-state fMRI study of the amygdala has considered sleep timing. In this study, similar to our own findings, weekend sleep midpoint was associated with altered connectivity between the amygdala and postcentral gyrus in children and adolescents (54). The postcentral gyrus is associated with processing somatosensory input (91). Though directionality varies, amygdala-postcentral gyrus connectivity has been associated with emotion regulation and externalizing symptoms (56). The paracingulate gyrus has previously been associated with response selection. Altered connections between this region and the amygdala could suggest that there are motor control differences in responses involving emotion for children with later sleep timing.

Later sleep timing may indicate that sleep timing is out of synchrony with circadian rhythms in children. Initiating sleep later than the optimal circadian phase, regardless of sleep duration, has been linked with increased risk for negative health outcomes in adults (92) and children (28). These associations may be because circadian phase shifts reduce sleep quality and lead to sleep problems (e.g., longer sleep onset latencies).

### Socioeconomic factors, sleep duration and timing, and amygdala connectivity

Socioeconomic disadvantage was indirectly associated with altered amygdala connectivity in these circuits via shorter weekday sleep duration and later weekend sleep midpoint. Socioeconomic context is theorized to be a distal factor that influences children’s health and development through multiple mediating mechanisms (2). Findings from this study provide some support for our proposal that sleep factors play a role in these mechanisms (20). Reduced sleep health may be part of the processes linking socioeconomic disadvantage with altered amygdala rsFC and in turn altered emotional functioning in children.

Prevention and intervention programs targeting sleep health during childhood may support brain development and emotional well-being. Components of effective interventions may include promoting recommended sleep hygiene practices to families and providing material resources to improve children’s sleep environments (93, 94). Sleep midpoint may be shifted earlier by implementing earlier bedtimes. This change may reduce misalignment between the child’s bedtime and circadian phase (21), which may support brain development. Advocating for cash supplements to families and neighborhood and housing policies that facilitate more optimal sleeping environments (e.g., reduced noise levels, light exposure, crowding) would help ensure that children across a range of socioeconomic backgrounds have support for sleep health (95).

### Strengths and limitations

Among this study’s strengths are its socioeconomically diverse sample, rigorous fMRI methods and control for head motion, and focus on multiple sleep parameters. In fMRI research, excessive motion can introduce significant noise and artifacts, making it difficult to distinguish genuine neural signal from confounds (75, 79). Children tend to exhibit greater motion compared to adults, emphasizing the necessity of motion correction to ensure the validity and interpretability of neuroimaging results in pediatric populations (80). Our sensitivity analyses confirmed the robustness of the results even when using strict exclusionary criteria for head motion.

This study also has limitations that should be considered when interpreting the results. First, parent reports were used to measure children’s sleep timing and duration. Although subjective assessment of sleep has strengths as a method, it can be biased (31, 96, 97). It would be valuable to replicate these findings using objective sleep measures, such as actigraphy, coupled with sleep diaries. Second, given the cross-sectional and correlational study design, it is not possible to infer causality. We also cannot rule out genetic influences on circadian preference. Bidirectional associations between sleep health and rsFC of the amygdala should be investigated in future longitudinal studies. Also, due to the cross-sectional design, the results of the mediation models should be interpreted with some caution (98). Third, the sample size was small, as many childhood fMRI samples are, and replication with a larger sample size would strengthen these findings. A crucial future direction is to examine the effects of exposure to racial/ethnic discrimination on sleep health in children (30). Exposure to racial/ethnic discrimination may increase stress, which interferes with sleep, and research is needed that disentangles the unique effects of socioeconomic disadvantage and racial/ethnic discrimination on children’s sleep health (99).

## Conclusion

To date, few studies of sleep and neural function have been equipped to address the role of socioeconomic context during childhood, which may reflect an important source of environmental influence on sleep quantity and quality. This study contributes to filling this gap in the literature. Findings indicated that socioeconomic disadvantage was associated with shorter sleep duration and later sleep timing, which made unique contributions to amygdala connectivity in emotion processing and regulation circuitry. Socioeconomic disadvantage in childhood can exert effects on mental health that persist into adulthood, and these effects may derive in part from how socioeconomic disadvantage interferes with sleep health during childhood. Insufficient sleep duration and quality during childhood may impact amygdala connectivity during sensitive periods of brain development, leading to enduring effects on mental health. Prevention and intervention strategies may need to evaluate and target children’s sleep sufficiency to support brain development more effectively. Bolstering neighborhood and family economic conditions could improve family routines and sleeping environments for children, helping to ensure that children across the socioeconomic spectrum have support for sleep health.

## Data availability statement

The raw data supporting the conclusions of this article will be made available by the authors, without undue reservation.

## Ethics statement

The studies involving humans were approved by Teachers College, Columbia University and New York State Psychiatric Institute. The studies were conducted in accordance with the local legislation and institutional requirements. Written informed consent for participation in this study was provided by the participants’ legal guardians/next of kin.

## Author contributions

MH: Conceptualization, Formal analysis, Methodology, Visualization, Writing – original draft, Writing – review & editing. KS: Methodology, Writing – review & editing. XH: Formal analysis, Methodology, Writing – review & editing. NS: Methodology, Writing – original draft, Writing – review & editing. MT: Methodology, Writing – review & editing. KN: Funding acquisition, Methodology, Supervision, Writing – review & editing. EM: Conceptualization, Formal analysis, Funding acquisition, Methodology, Supervision, Writing – original draft, Writing – review & editing.
