# Supplementary material for: Socioeconomic factors, sleep timing and duration, and amygdala resting-state functional connectivity in children
Source: Front Psychiatry. 2024 May 22;15:1373546. doi: 10.3389/fpsyt.2024.1373546 (PMC11150855; doi:10.3389/fpsyt.2024.1373546)
Supplement: Supplementary file 1 [file DataSheet_1.docx]

**Supplemental Material**

**Table S1. Zero-order correlations between socioeconomic factors and sleep characteristics** **(*N* = 92)**

|  |  | 1 | 2 | 3 | 4 | 5 | 6 | 7 | 8 | 9 | 10 |
| --- | --- | --- | --- | --- | --- | --- | --- | --- | --- | --- | --- |
| 1 | Family income-to-needs ratio | -- |  |  |  |  |  |  |  |  |  |
| 2 | Parental education | .68*** | -- |  |  |  |  |  |  |  |  |
| 3 | Weekday bedtime | -.34*** | -.38*** | -- |  |  |  |  |  |  |  |
| 4 | Weekday wake-up time | -.10 | -.07 | .29** | -- |  |  |  |  |  |  |
| 5 | Weekday sleep midpoint | -.29** | -.29** | .83*** | .78*** | -- |  |  |  |  |  |
| 6 | Weekday sleep duration | .22* | .29** | -.66*** | .52*** | -.13 | -- |  |  |  |  |
| 7 | Weekend bedtime | -.34** | -.59*** | .58*** | .07 | .43*** | -.47*** | -- |  |  |  |
| 8 | Weekend wake-up time | -.29** | -.46*** | .39*** | .20^+^ | .38*** | -.19^+^ | .57*** | -- |  |  |
| 9 | Weekend sleep midpoint | -.35*** | -.59*** | .54*** | .16 | .45*** | -.36*** | .86*** | .91*** | -- |  |
| 10 | Weekend sleep duration | -.02 | .03 | -.10 | .17 | .03 | .22* | -.30** | .62*** | .23* | -- |

^+^*p* < .10, **p* < .05, ***p* < .01, ****p* < .001

**
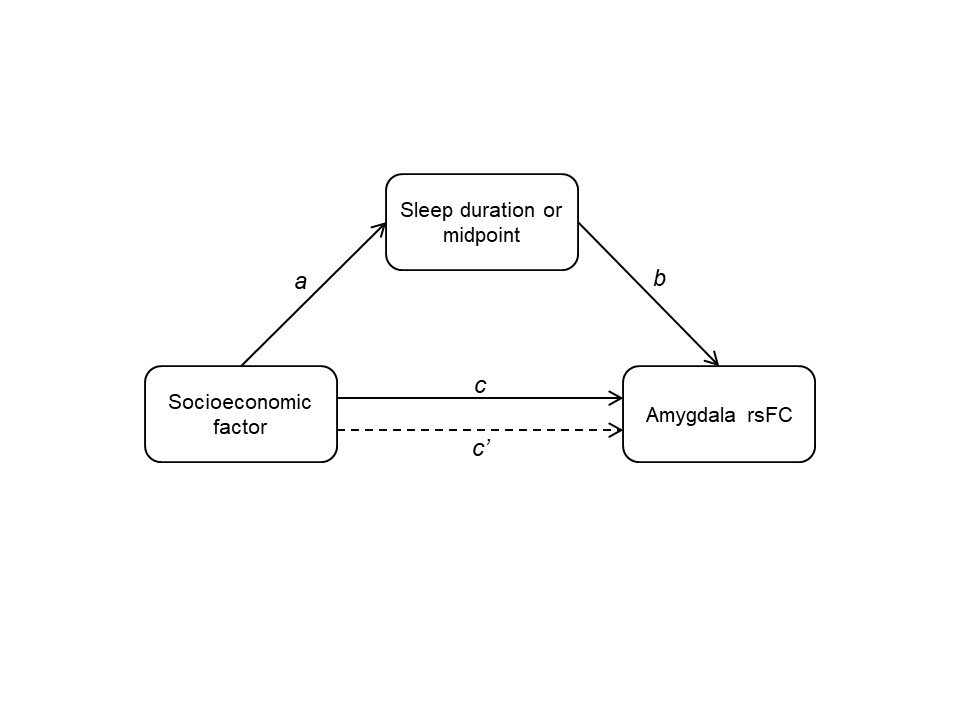
**

**Figure S1.** Hypothesized mediation model. The *a* path is an estimation of the association between the independent variable and the mediator. The *b* path is an estimation of the association between the mediator and outcome, controlling for the independent variable. The *c* path represents the total effect, and the *c’* path represents the direct effect after accounting for the indirect effect. Socioeconomic factors were family income-to-needs ratio and parental education.

rsFC, resting-state functional connectivity

**a)**


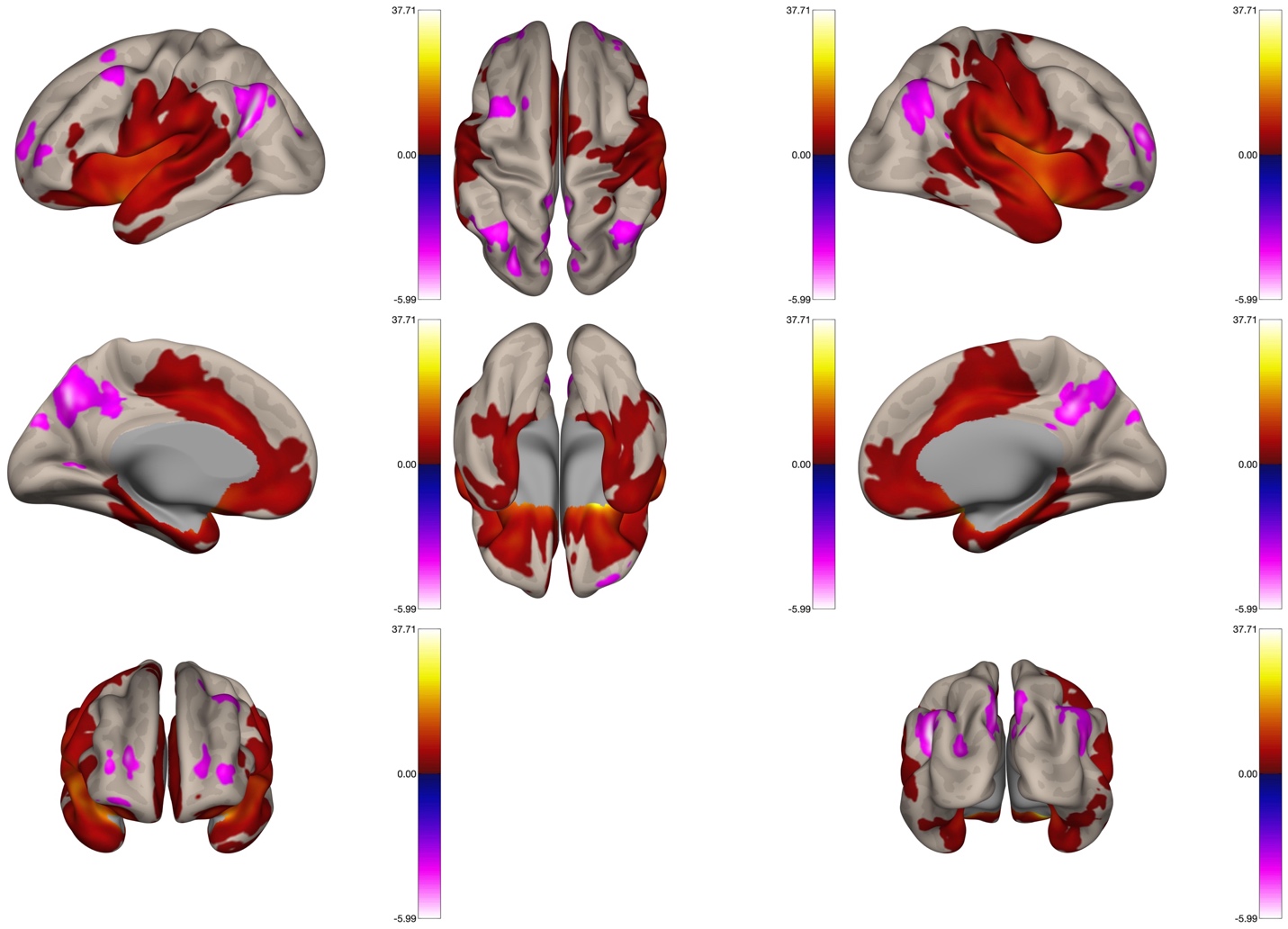

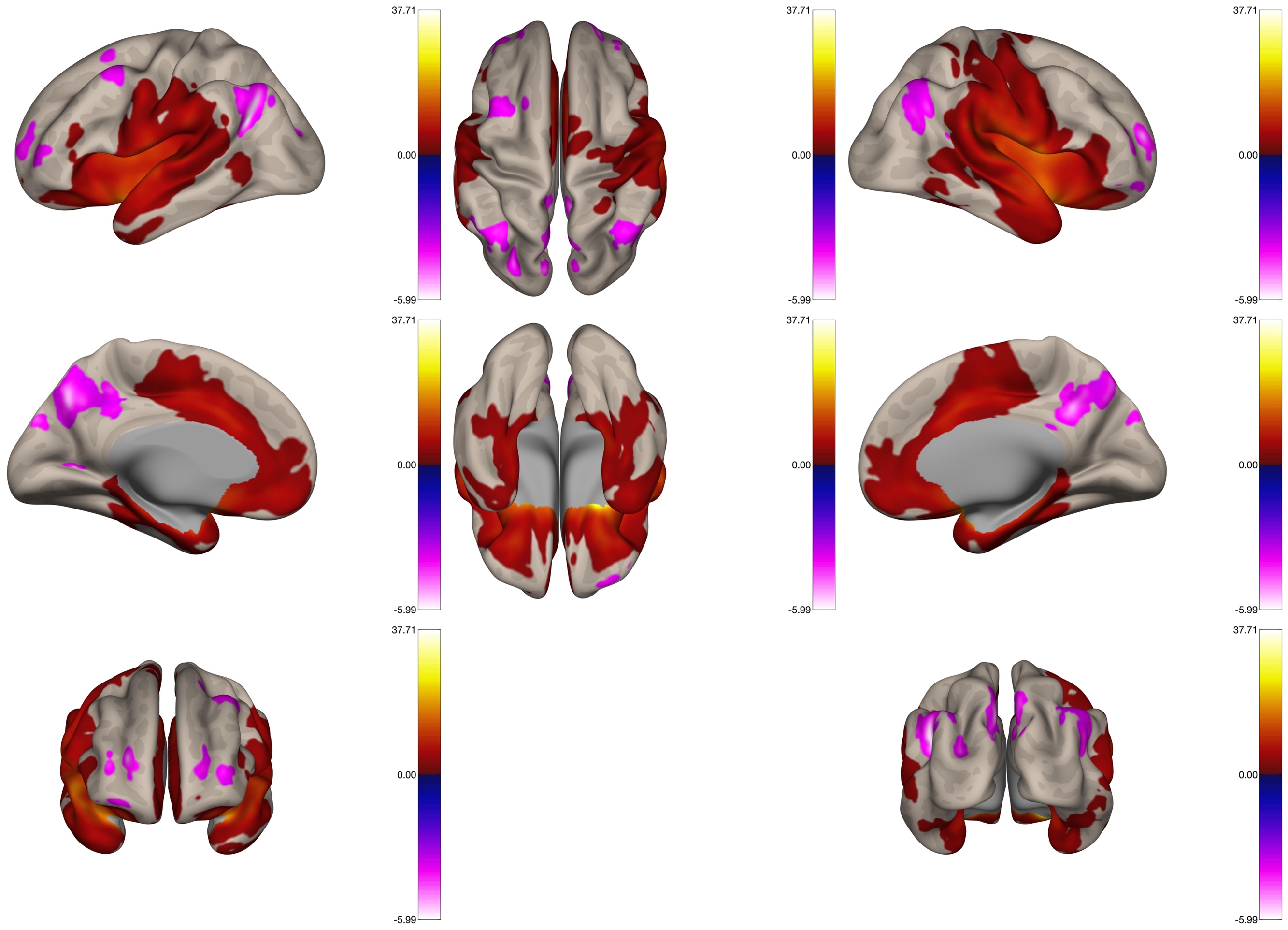

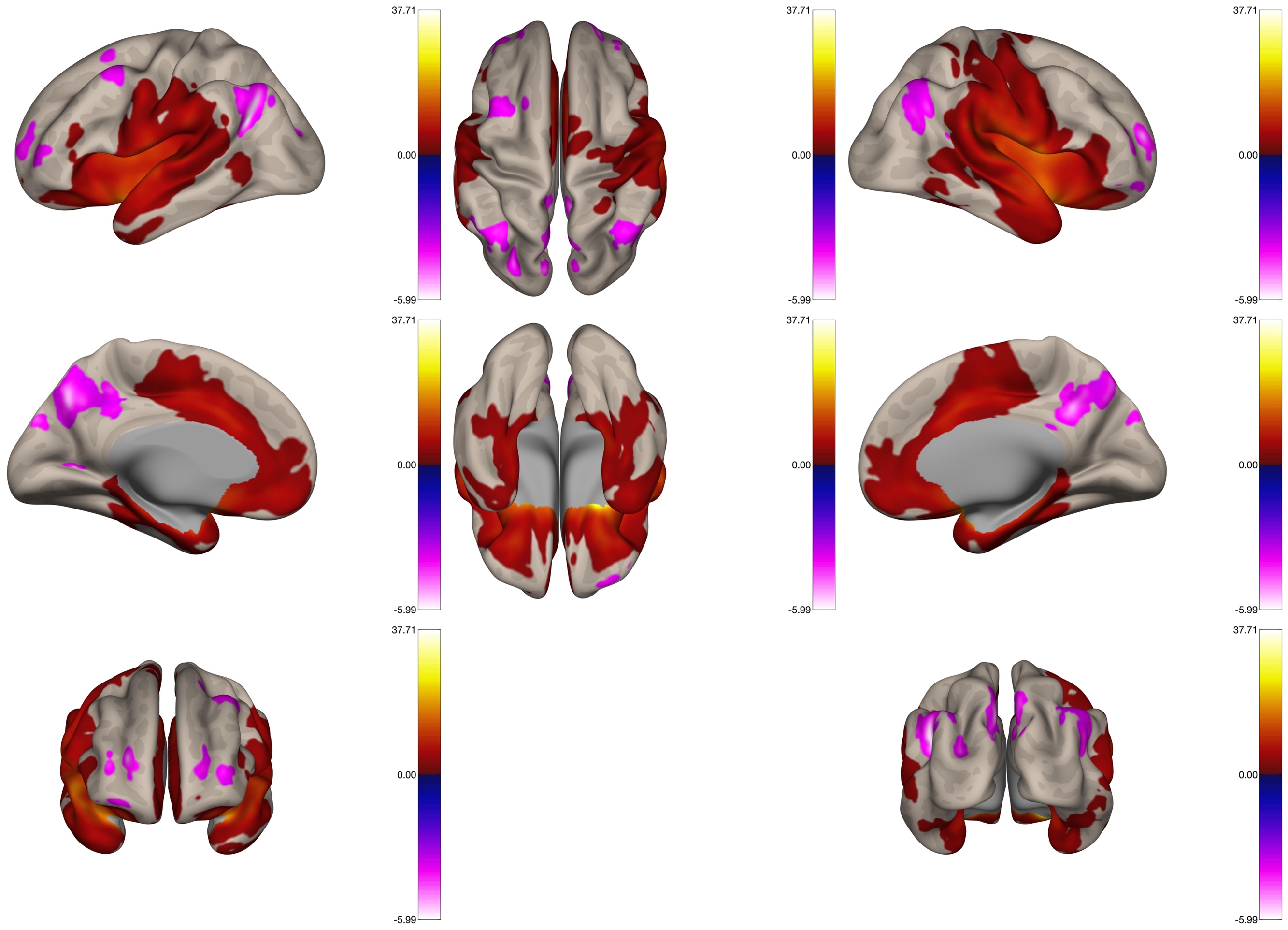

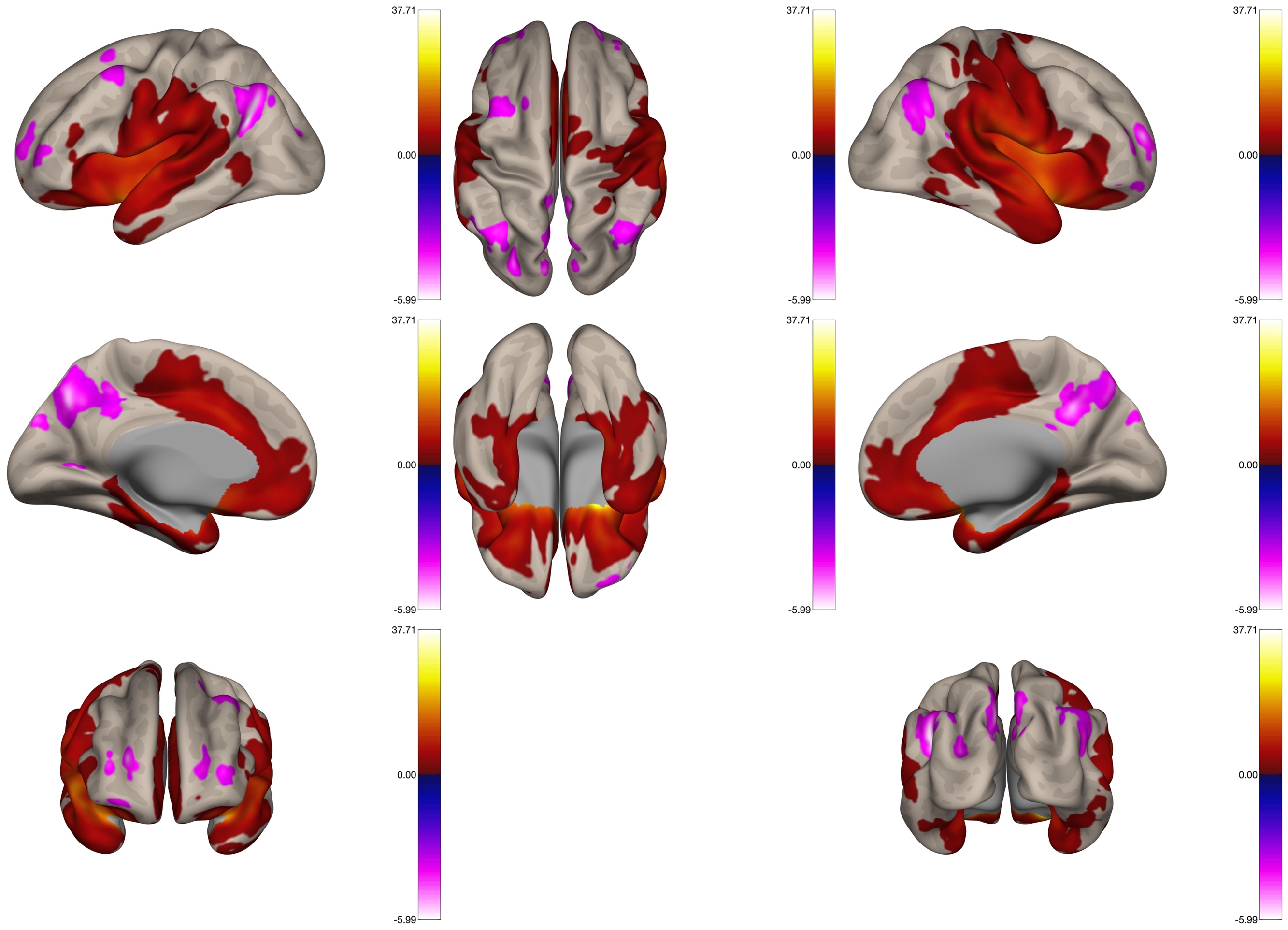

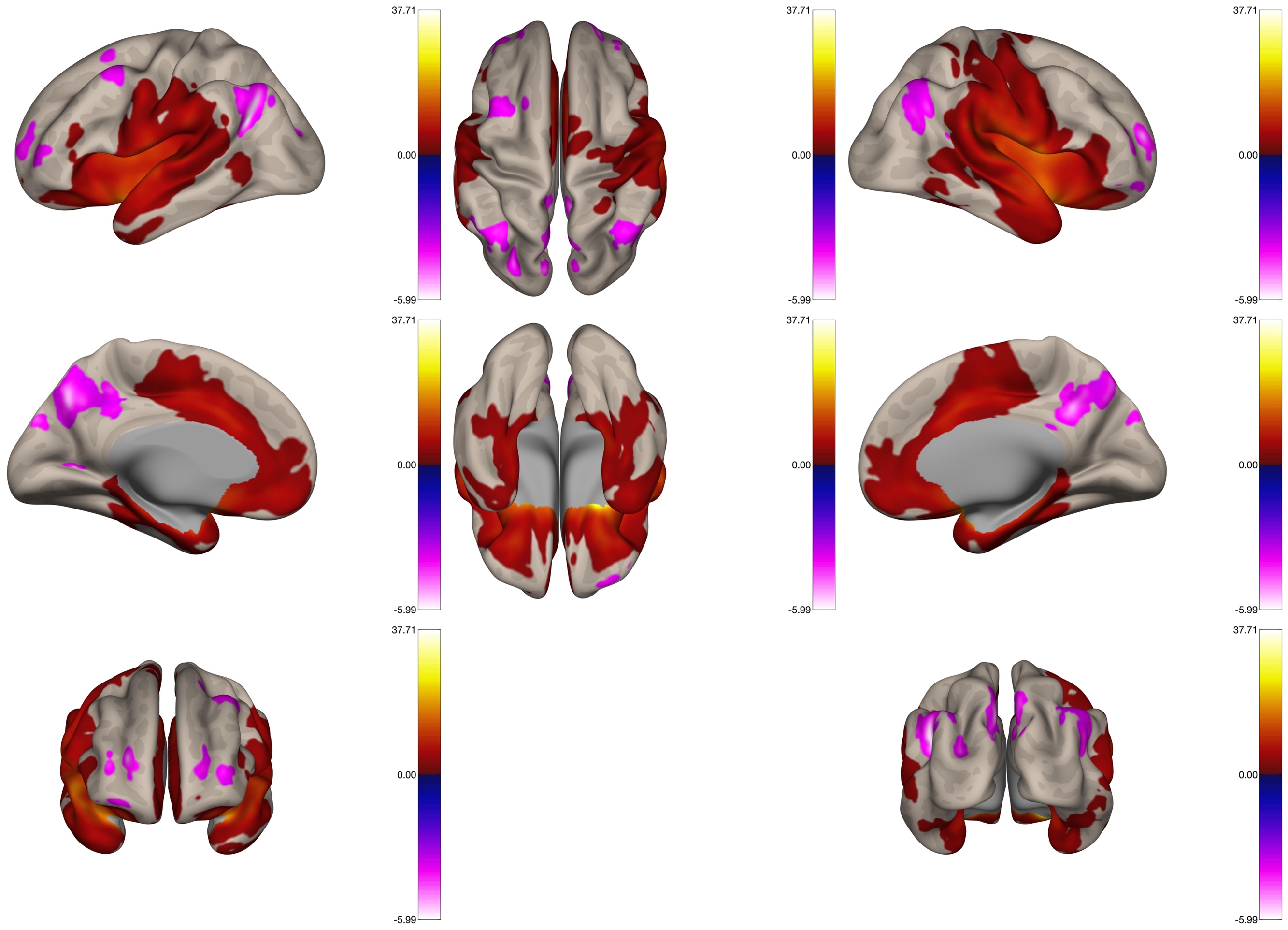


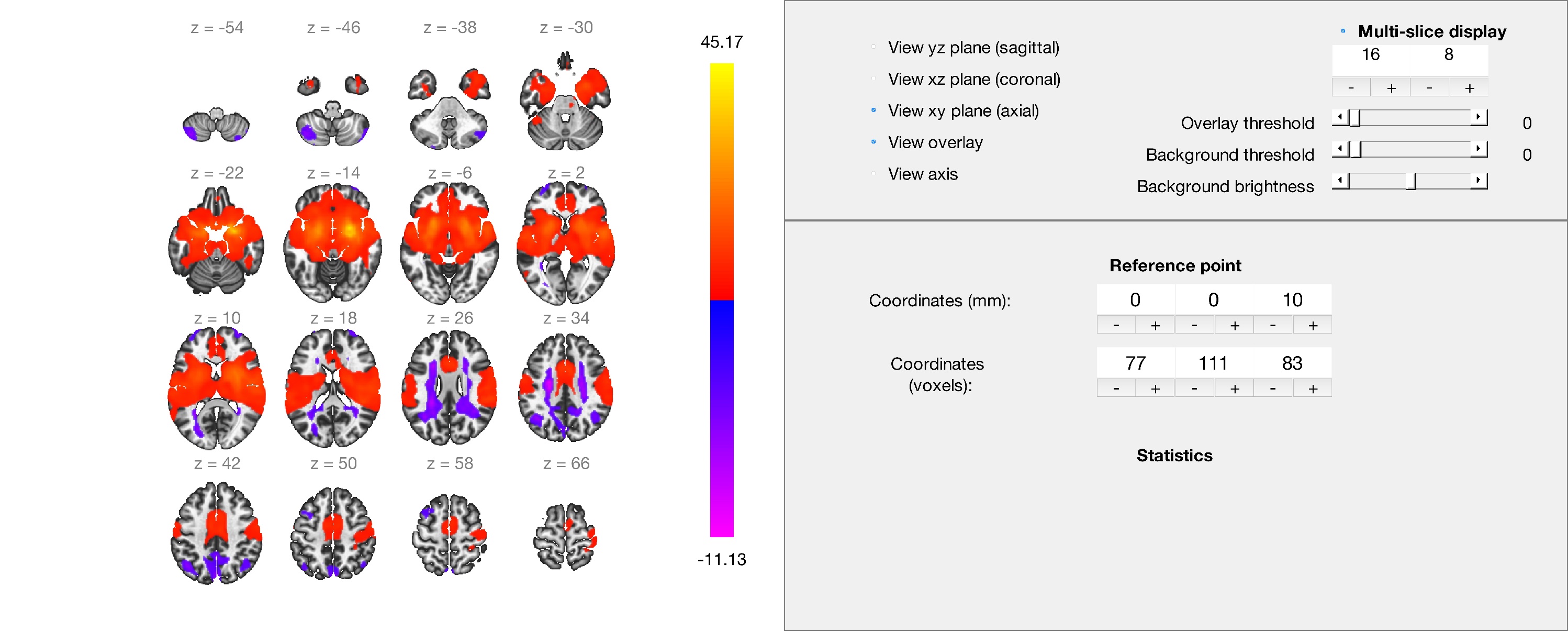

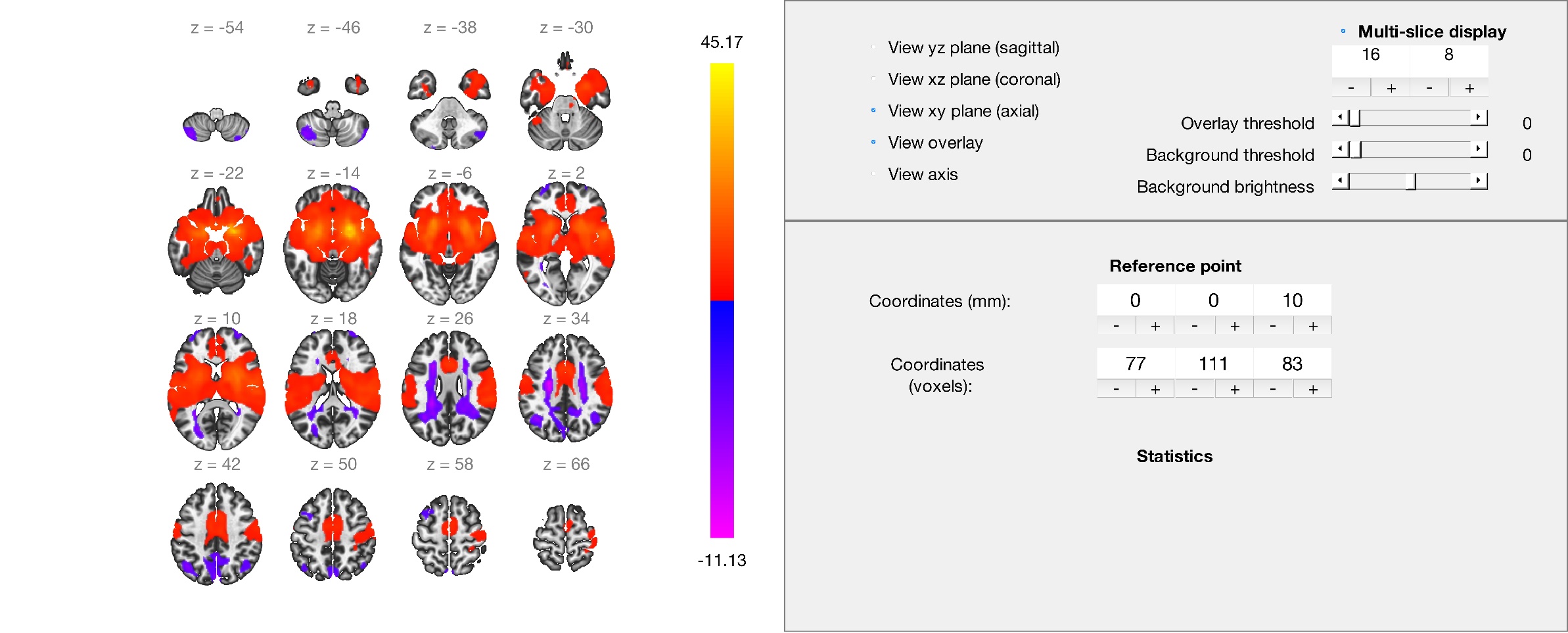


**
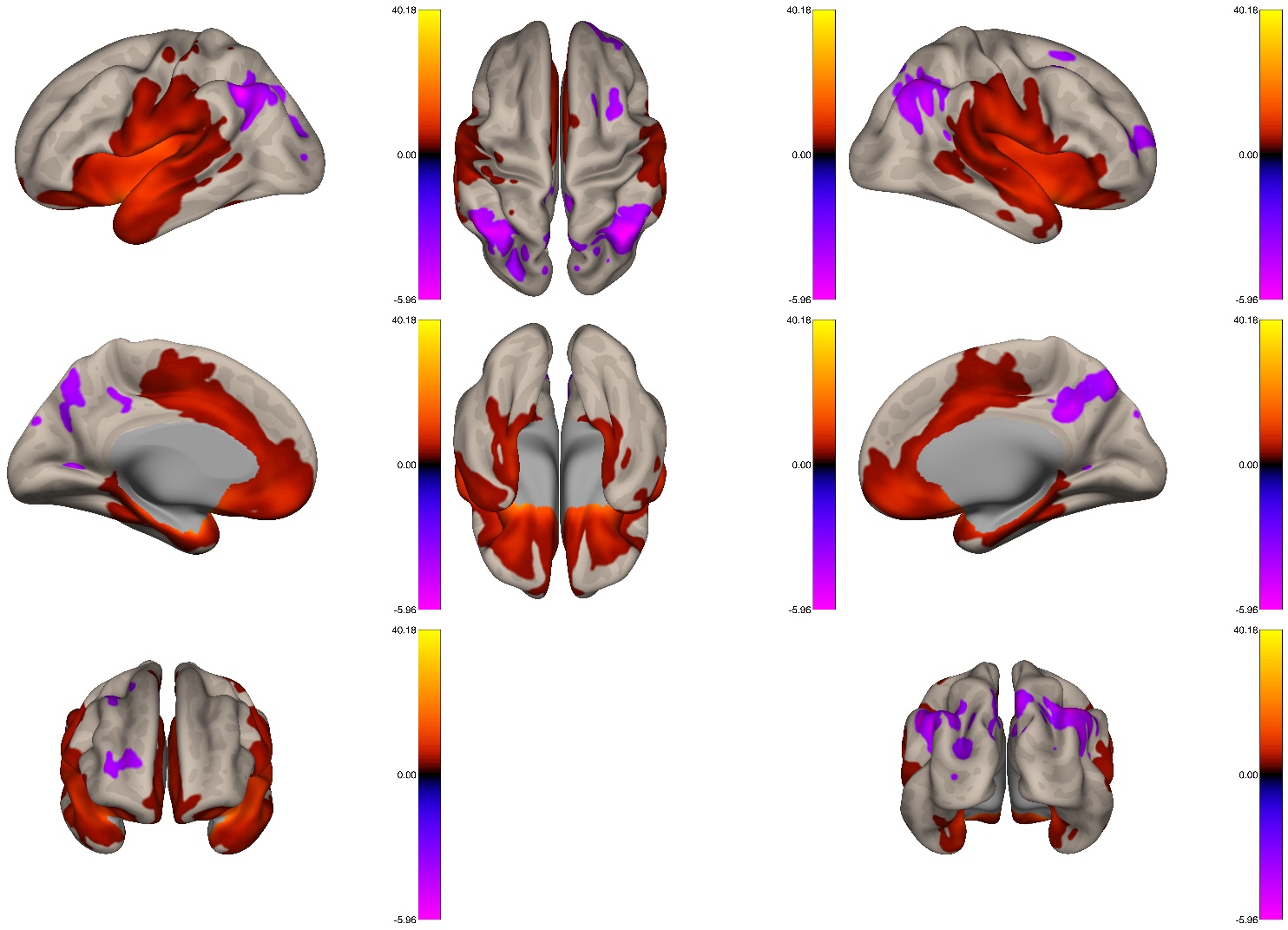

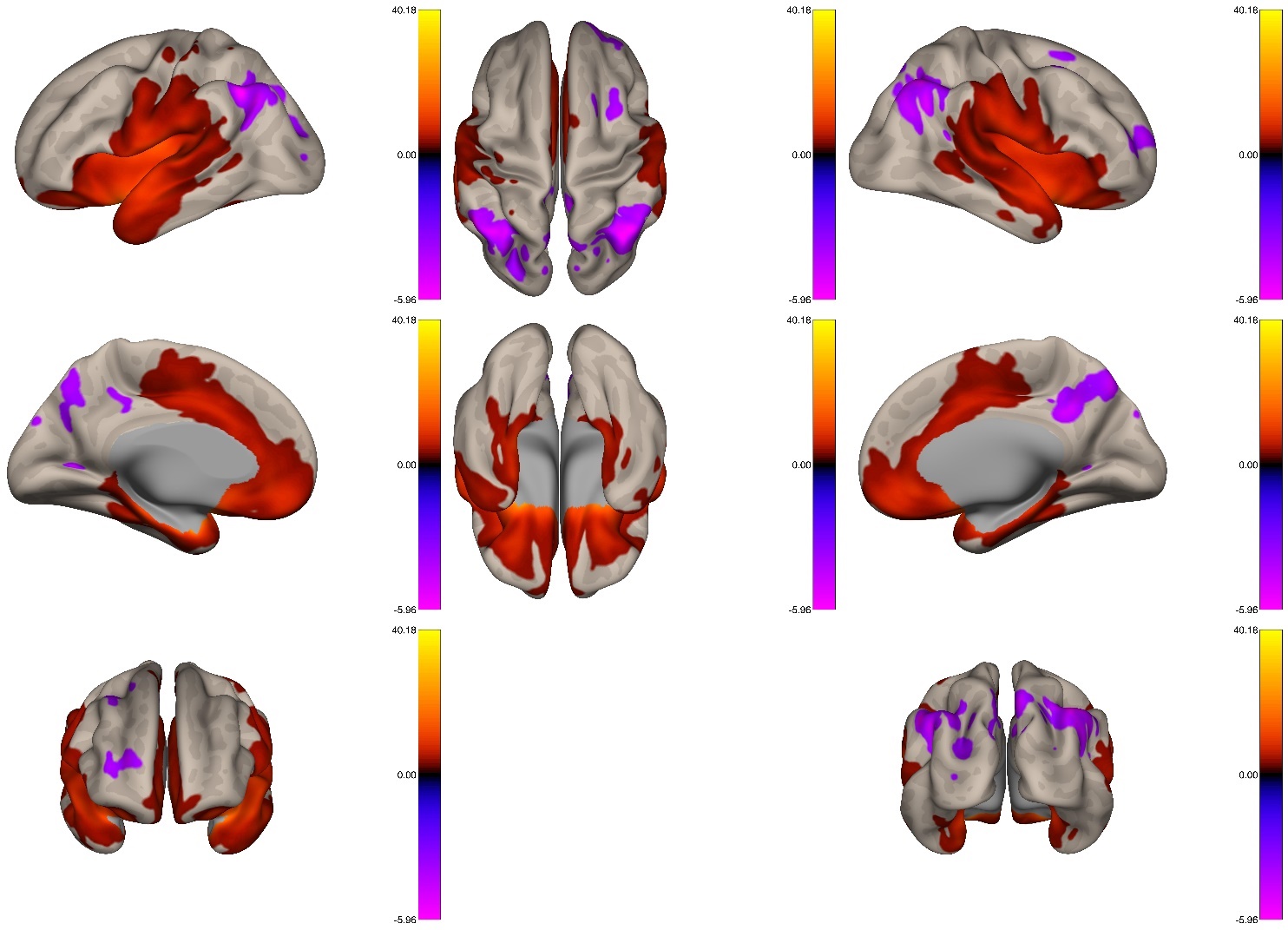
b)**


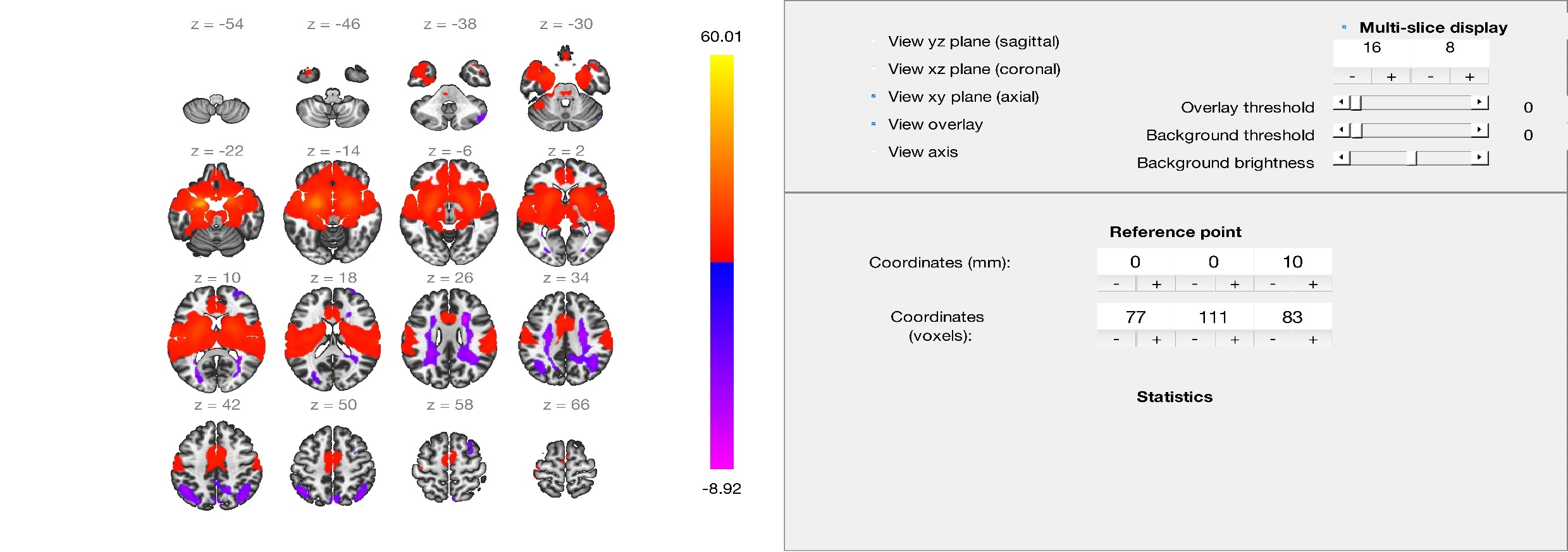

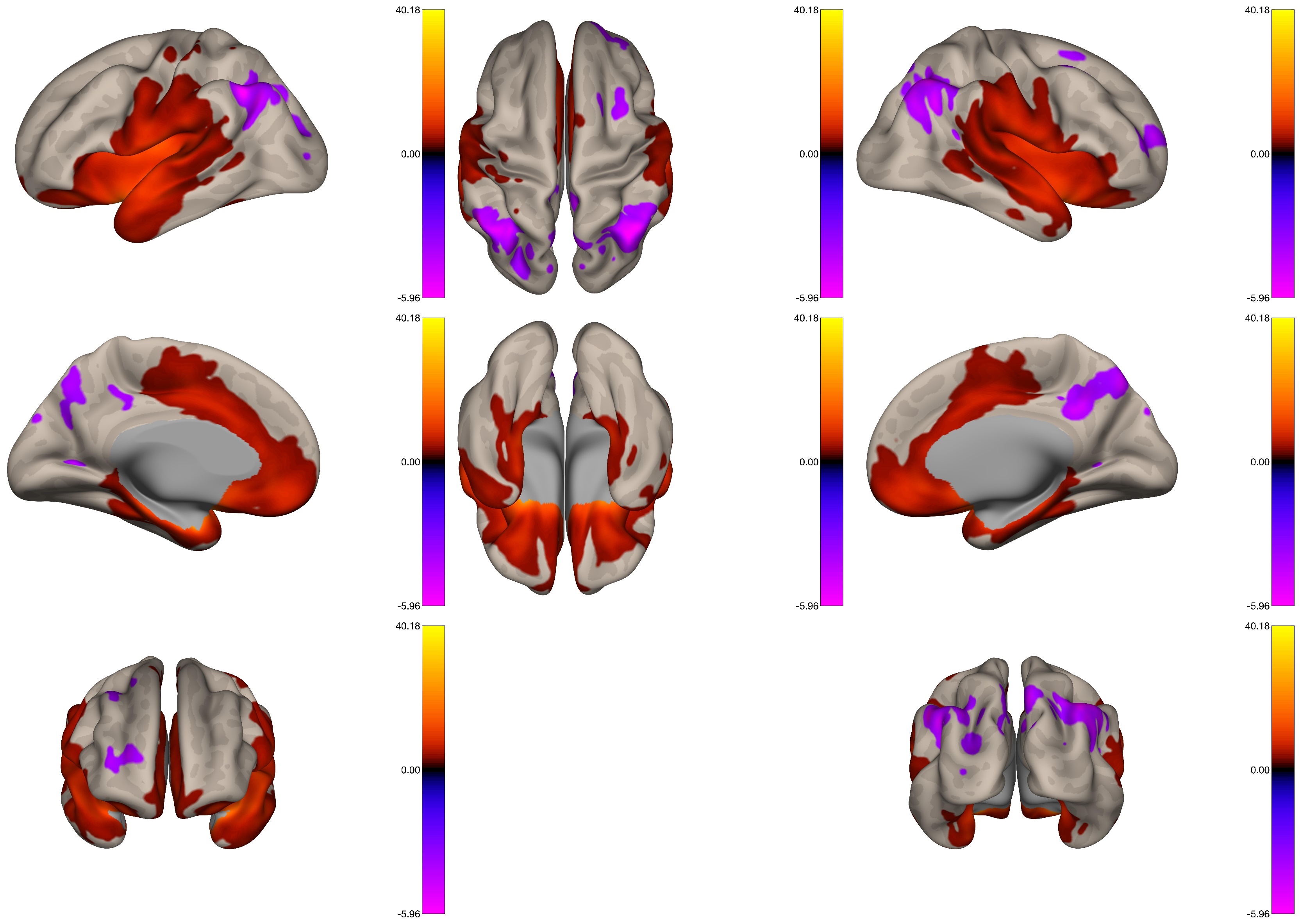


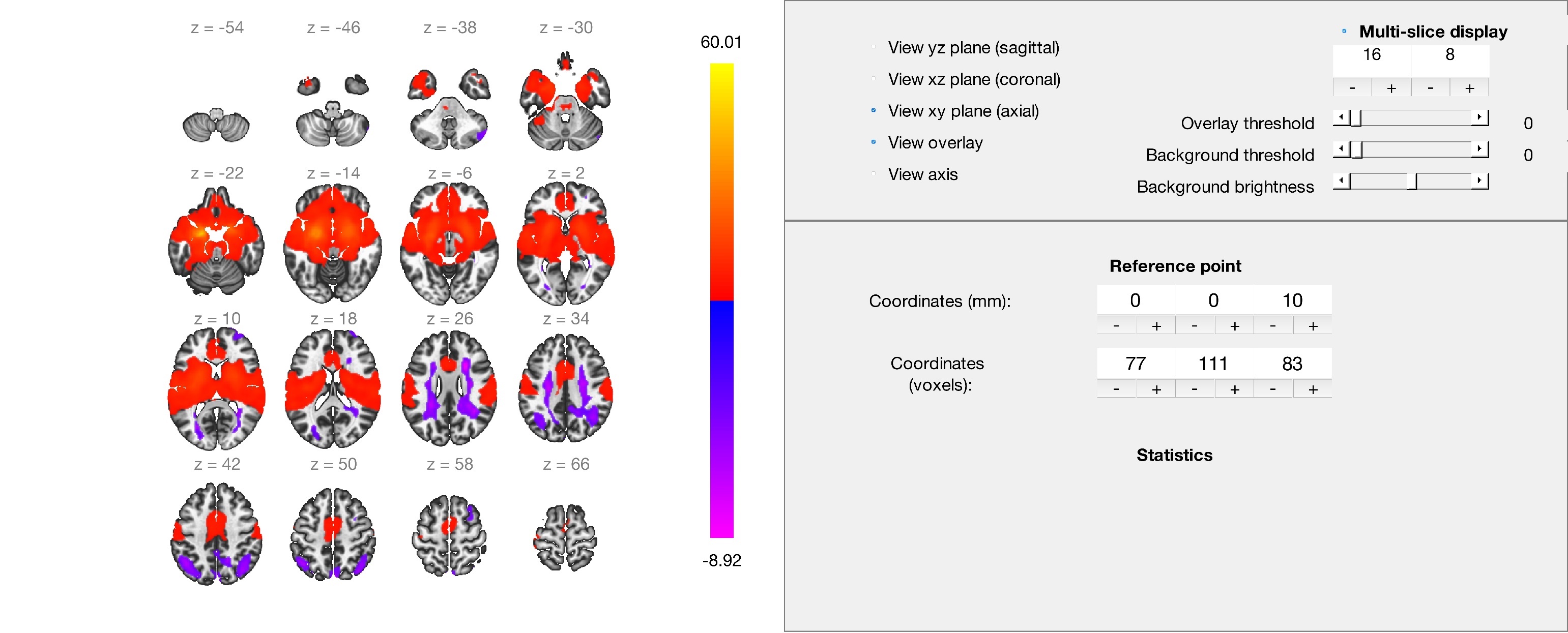


**Figure S2.** On average, children were found to exhibit widespread positive (**a)** left amygdala and (**b)** right amygdala resting-state functional connectivity. Models were corrected for multiple comparisons; cluster threshold: *p* < 0.05 cluster size p-FDR corrected; voxel threshold: *p* < 0.001 (uncorrected).

**a)**

**
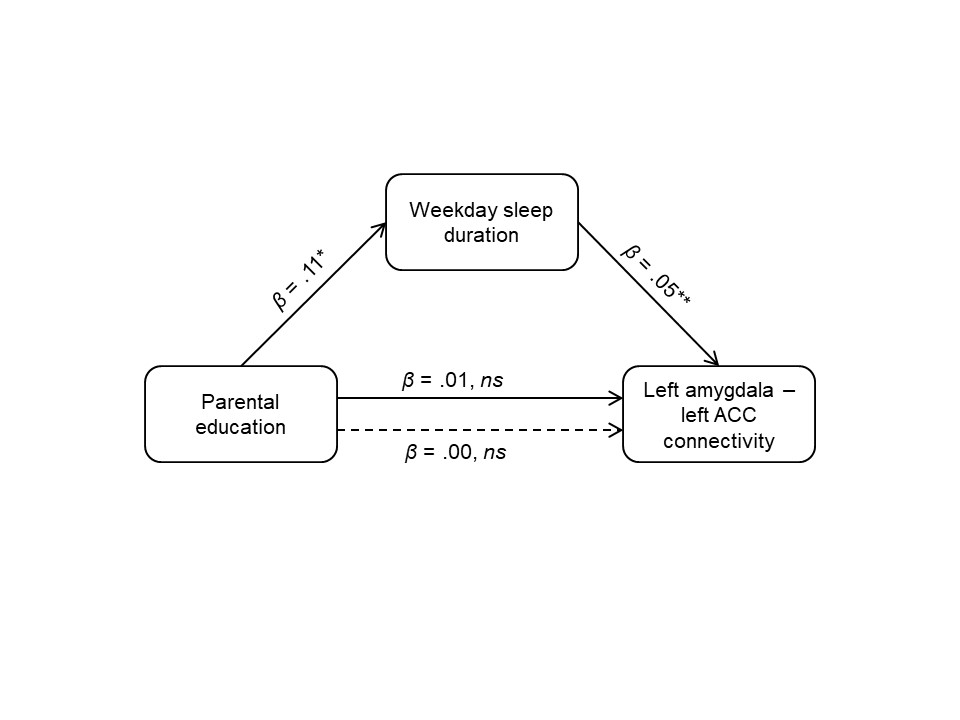
**

**b)**

**
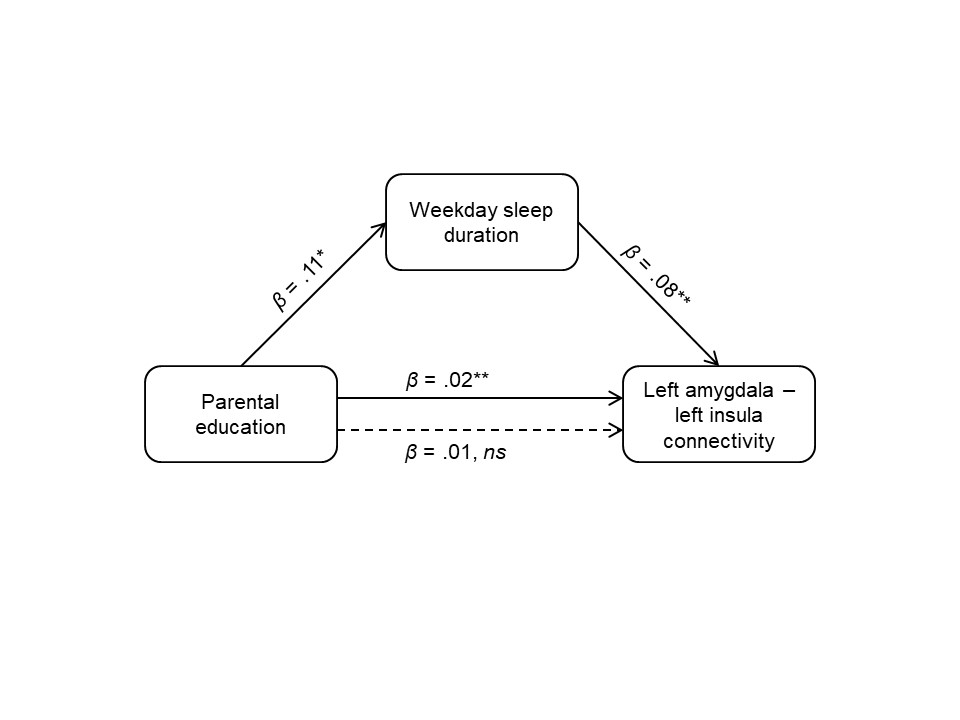
**

**Figure S3**. Parental education was indirectly associated with (**a)** left amygdala-left anterior cingulate cortex (ACC) connectivity and (**b)** left amygdala-left insula connectivity via weekday sleep duration in children. Each solid line between parental education and amygdala connectivity shows the total effect (*c* path), while the dotted line shows the direct effect after accounting for the indirect effect (*cʹ* path). **p* < .05, ***p* < .01

**a)**

**
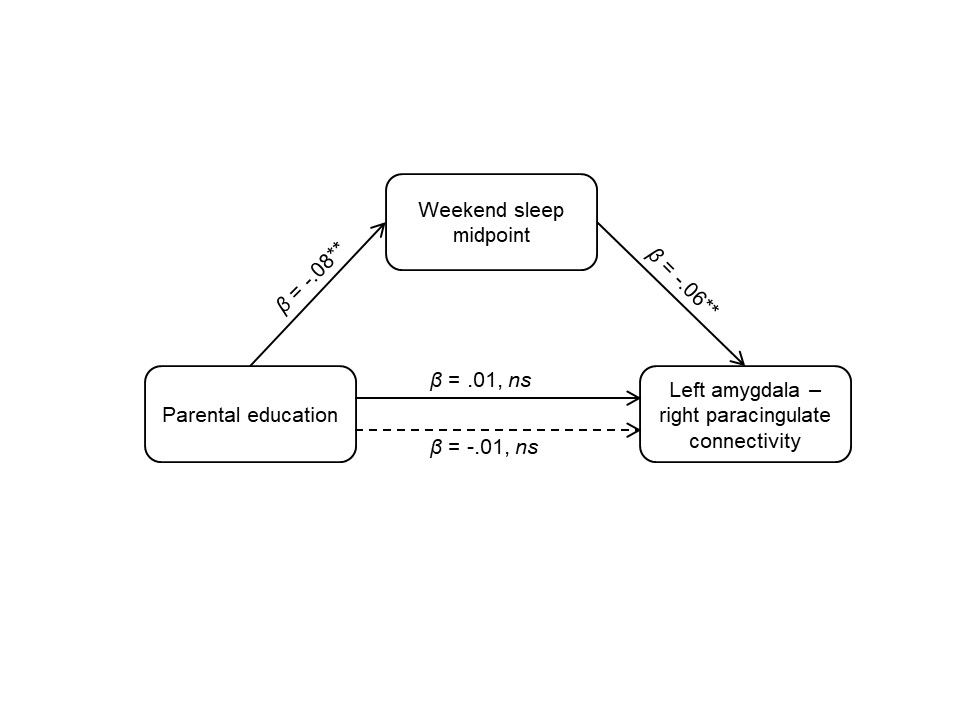
**

**b)**

**
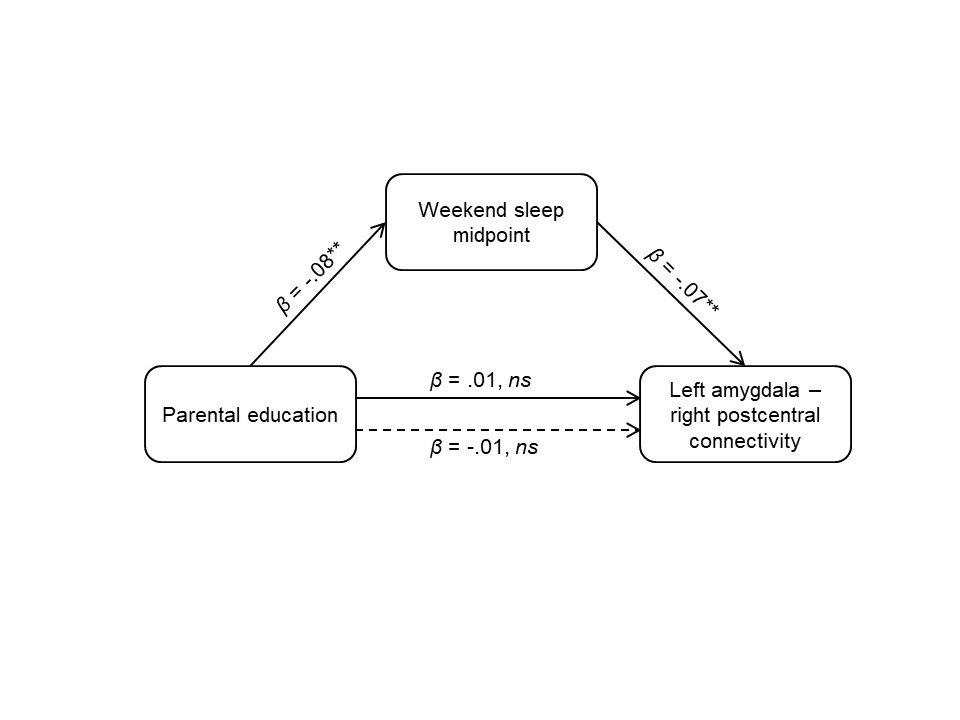
**

**Figure S4**. Parental education was indirectly associated with (**a)** left amygdala—right paracingulate cortex connectivity and (**b)** left amygdala-right postcentral gyrus connectivity via weekend sleep midpoint in children. Each solid line between parental education and amygdala connectivity shows the total effect (*c* path), while the dotted line shows the direct effect after accounting for the indirect effect (*cʹ* path). **p* < .05, ***p* < .01
